# Spike-Based Population Coding and Working Memory

**DOI:** 10.1371/journal.pcbi.1001080

**Published:** 2011-02-17

**Authors:** Martin Boerlin, Sophie Denève

**Affiliations:** 1 Group for Neural Theory, Département d'Études Cognitives, École Normale Supérieure, Paris, France; 2 Laboratoire de Neurosciences Cognitives, Inserm U960, Paris, France; University College London, United Kingdom

## Abstract

Compelling behavioral evidence suggests that humans can make optimal decisions despite the uncertainty inherent in perceptual or motor tasks. A key question in neuroscience is how populations of spiking neurons can implement such probabilistic computations. In this article, we develop a comprehensive framework for optimal, spike-based sensory integration and working memory in a dynamic environment. We propose that probability distributions are inferred spike-per-spike in recurrently connected networks of integrate-and-fire neurons. As a result, these networks can combine sensory cues optimally, track the state of a time-varying stimulus and memorize accumulated evidence over periods much longer than the time constant of single neurons. Importantly, we propose that population responses and persistent working memory states represent entire probability distributions and not only single stimulus values. These memories are reflected by sustained, asynchronous patterns of activity which make relevant information available to downstream neurons within their short time window of integration. Model neurons act as predictive encoders, only firing spikes which account for new information that has not yet been signaled. Thus, spike times signal deterministically a prediction error, contrary to rate codes in which spike times are considered to be random samples of an underlying firing rate. As a consequence of this coding scheme, a multitude of spike patterns can reliably encode the same information. This results in weakly correlated, Poisson-like spike trains that are sensitive to initial conditions but robust to even high levels of external neural noise. This spike train variability reproduces the one observed in cortical sensory spike trains, but cannot be equated to noise. On the contrary, it is a consequence of optimal spike-based inference. In contrast, we show that rate-based models perform poorly when implemented with stochastically spiking neurons.

## Author Summary

Most of our daily actions are subject to uncertainty. Behavioral studies have confirmed that humans handle this uncertainty in a statistically optimal manner. A key question then is what neural mechanisms underlie this optimality, i.e. how can neurons represent and compute with probability distributions. Previous approaches have proposed that probabilities are encoded in the firing rates of neural populations. However, such rate codes appear poorly suited to understand perception in a constantly changing environment. In particular, it is unclear how probabilistic computations could be implemented by biologically plausible spiking neurons. Here, we propose a network of spiking neurons that can optimally combine uncertain information from different sensory modalities and keep this information available for a long time. This implies that neural memories not only represent the most likely value of a stimulus but rather a whole probability distribution over it. Furthermore, our model suggests that each spike conveys new, essential information. Consequently, the observed variability of neural responses cannot simply be understood as noise but rather as a necessary consequence of optimal sensory integration. Our results therefore question strongly held beliefs about the nature of neural “signal” and “noise”.

## Introduction

Our senses furnish us with information about the external world that is ambiguous and corrupted by noise. Taking this uncertainty into account is crucial for a successful interaction with our environment. Psychophysical studies have shown that animals and humans can behave as optimal Bayesian observers, i.e. they integrate noisy sensory cues, their own predictions and prior beliefs in order to maximize the expected outcome of their actions [1], [2], [3], [4].

Several theoretical investigations have explored the neural mechanisms that could underly such probabilistic computations [5], [6], [7], [8], [9], [10]. In cortical areas, sensory and motor variables are encoded by the joint activity of populations of spiking neurons [11], [12] whose activity is highly variable and weakly correlated [13], [14]. The timing of individual spikes is unreliable while spike counts are approximately Poisson distributed [14]. These characteristics have inspired rate-based models that encode probability distributions in their average firing rates and spike count covariances. Previous studies have examined analytically and empirically how this information can be encoded in a population code [6], [5], [15], [10], [9], [16], [17], [18], how it can be decoded [19], [20], [5], [21], [10], [22], [23], [24] and how population codes can be combined optimally [6], [25]. In particular, optimal cue combination reduces to a simple linear combination of neural activities for a broad family of neural variability, including Poisson or Gaussian noise [6].

However, most of these studies neglect a crucial dimension of perception: time. Most sensory stimuli vary dynamically in a natural environment, which requires sensory representations to be constructed, integrated and combined on-line [23], [21]. Perceptual inference thus cannot be based on rates or spike counts measured during a “fixed” temporal window, as used in most previous population coding frameworks. At the same time, reliable decisions typically require an integration of sensory evidence over hundreds of milliseconds [26], [27], which largely exceeds the integrative time constant of single neurons. It is unclear how such leaky devices could compute sums of spike counts on the typical time scale of perceptual or motor tasks.

The problem is even more crucial if the decision is delayed compared to the presentation of sensory information. Sensory variables such as the direction of motion of a stimulus can be retained in “working memory” for significant periods of time even in the absence of sensory input. Neural correlates of this working memory appear as persistent neural activity in parietal and frontal brain areas and exhibit firing statistics similar to those found for sensory responses [28],[27],[29]. This persistent activity has been modeled as a stable state of recurrent neural network dynamics [30]. However, such attractors correspond to stereotyped patterns of activity that can only represent a single stimulus value. For example, the memorized position of an object can be encoded by the position of a stable “bump” of activity [30], [31]. This would imply though that information about the reliability of the memorized cue is lost and cannot be used for delayed cue combination or decision making. We hypothesize instead that stimuli are memorized in the same format as sensory input, i.e. as a probability distribution. The question of how probability distributions can be memorized by a population of neurons remains largely unanswered.

Here, we approach these issues by using a new interpretation of population coding in the context of temporal sensory integration. We consider spikes, rather than rates, as the basic unit of probabilistic representation. We show how recurrent networks of leaky integrate-and-fire neurons can construct, combine and memorize probability distributions of dynamic sensory variables. Spike generation in these neurons results from a competition between an integration of evidence from feed-forward sensory inputs and a prediction from lateral connections. A neuron therefore acts as a “predictive encoder”, only spiking if its input cannot be predicted by its own or its neighbors' past activity.

We demonstrate that such networks integrate and combine sensory inputs optimally, i.e. without losing information, and track the stimulus dynamics spike-per-spike even in the absence of sensory input, over timescales much longer than the neural time constants. This framework thus provides a first comprehensive theory for optimal *spike-based* sensory integration and working memory. In contrast to rate models implemented with Poisson spiking neurons, this model does not require large levels of redundancy to compensate for the noise added by stochastic spike generation.

Similar to cortical sensory neurons, model neurons respond with sustained, asynchronous spiking activity. Spike times are variable and uncorrelated, despite the deterministic spike generation rule. However, in contrast to rate codes, each spike “counts”. The trial to trial variability of spike trains does not reflect an intrinsic source of noise that requires averaging, but is a consequence of predictive coding. While spike times are unpredictable at the level of a single neuron, they deterministically represent a probability distribution at the level of the population. This leads us to reinterpret the notions of signal and noise in cortical neural responses.

## Results

### Goal of the model

In order to clarify the presentation, we will concentrate on the following general task. Imagine a cat chasing a mouse in your garden. The cat integrates auditory and visual information to locate the mouse. It will combine these cues according to their reliability. If for instance the mouse is partially covered by a bush, i.e. there is a high uncertainty associated with the visual cue, the cat will give a higher weight to its auditory information. If the mouse suddenly disappears behind a tree and cannot be heard or seen anymore, the cat should estimate the likely trajectory of the mouse in the absence of any relevant sensory input, in order to anticipate where the mouse is going to reappear. Finally, this information will need to be extracted when the cat eventually decides to catch the mouse.

**Figure 1:**
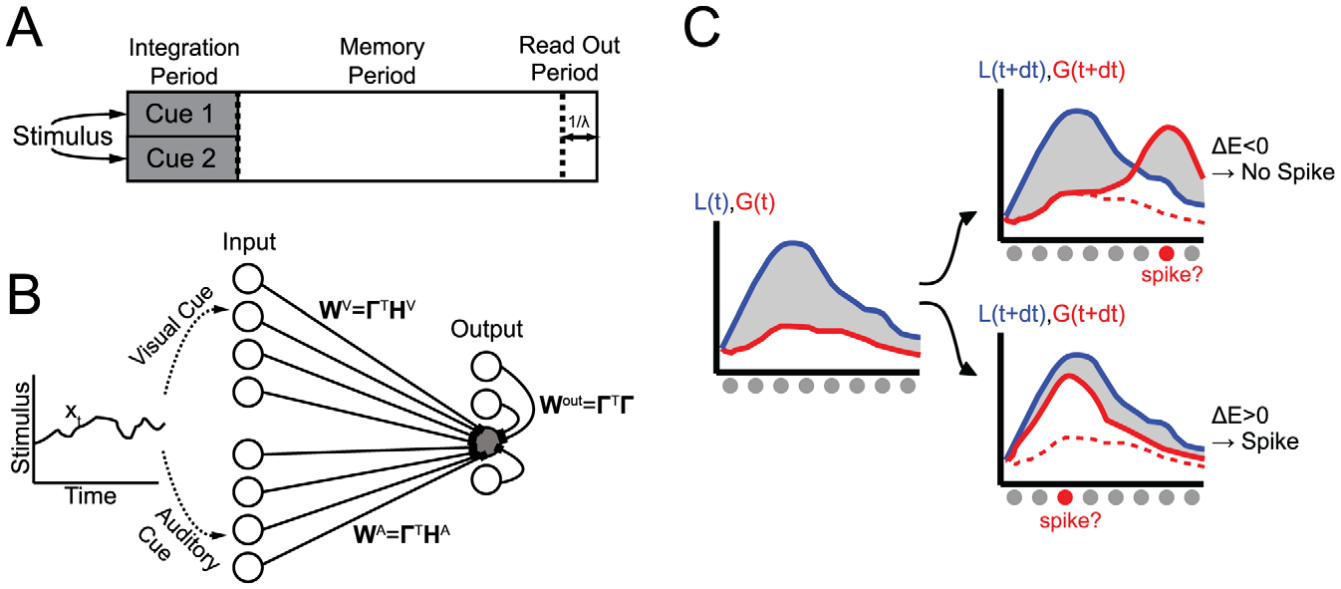
Illustrations. (A) Illustration of the network task. An auditory and a visual cue (cue 1 and 2) about a dynamic stimulus (e.g. the position of a mouse) are integrated and combined during the integration period. During the memory period, this information is kept available such that it can be read out over a timescale of order 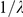 during the read-out period. (B) Schematic illustration of the network. The visual and the auditory cue about stimulus 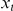 are encoded in two independent input populations that send feed-forward inputs to the output population. The output population is recurrently connected. The connection weights 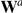 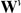 and 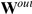 are functions of the input kernels 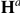 and 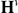 as well as the output kernel 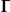. (C) Illustration of the spike generation rule. 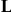 denotes the stimulus posterior given all inputs and 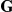 represents an approximation to 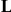 that is decoded from the output spike trains. 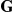 should be as close as possible to 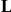. An output spike adds a kernel to 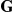. If its effect is to reduce the mean squared distance between the curves (down right), the spike is fired. The spike is not generated however if it increases the distance between the two curves (top right).

The cat's task can thus be divided into three parts (figure 1A). First, during a sensory integration period, sensory cues about a dynamic stimulus, 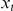, are combined over modalities and time in order to get a more refined estimate about the stimulus. Second, during a memory period, the evolution of the stimulus is predicted and tracked while past information is kept available. Finally, during a decoding period, the position of the mouse is extracted from the memorized information.

We assume that the dynamic stimulus 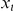 evolves according to a drift-diffusion process of the form
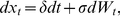
(1)where 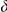 and 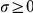 are parameters and 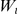 is a Wiener process. The first term on the right-hand side of equation (1) describes the predictable drift of the stimulus. Intuitively, it describes the velocity of the stimulus. The second term describes stochastic and therefore unpredictable changes in the stimulus. This is the diffusive part of the stimulus dynamics.

Visual and auditory inputs are provided by two independent population of neurons on two input layers, a “visual” layer and an “auditory” layer. Input neurons respond to position 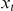 with noisy spike trains 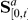 (auditory) and 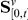 (visual). We denote 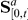 the auditory spike trains observed up to time t, and 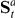 the number of spikes observed in a small temporal window 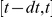 such that 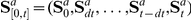. We assume that sensory input spikes depend instantaneously on the stimulus 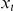 and are conditionally independent of the past, i.e. 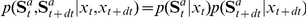. Moreover, we consider sensory likelihoods that belong to the exponential family of probability distributions with linear sufficient statistics. In this case, the log probability of observing 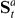 spikes in the auditory layer can be written as a sum of spike counts
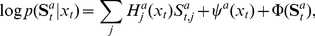
(2)where 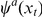 and 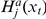 are functions of 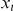 and 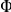 acts as a normalization term. We will refer to 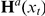 and 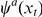 as the kernel and the bias of the auditory likelihood respectively. A similar equation holds for the visual likelihood. The family of distributions described by equation (2) captures most popular models of neural noise including Poisson noise, Gaussian or exponential noise, with or without correlations. In this article, we assume independent Poisson noise for simplicity. In this case, the kernels correspond to the log tuning curves, 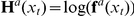 and 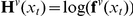, where 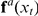 and 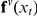 are the visual and auditory tuning curves (see Materials and Methods).

The two sensory input layers converge onto a recurrently connected output layer (figure 1B) that generates a set of output spike trains, 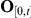. We want these output spikes to represent the posterior probability of the position of the mouse given the visual and auditory spike trains. For this purpose, we define an “on-line decoder”, 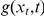, that reads out the information in the output population through a leaky integration of output spikes. The advantages of such a read-out function will be discussed shortly below. We define 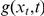 such that
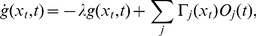
(3)where 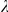 is a leak term, 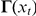 defines a choice of output kernels, and 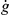 stands for the temporal derivative of 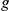. The network structure and dynamics shall ensure that this read-out approximates the log posterior of the combined inputs:
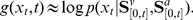
(4)


If this equation holds, the output neurons are said to encode the stimulus “optimally”.

This decoder defines how the posterior probability is represented on-line (i.e. within time constant 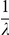) by the output spike trains. However, perceptual or motor tasks might never require an explicit read-out of probability distributions. The decoder is therefore a theoretical construct that does not have to be implemented in any specific neural structure.

The coding strategy for the output layer is chosen for self-consistency, i.e. it ensures that 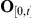 can be used as input for further processing stages. Indeed, 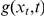 is treated as a log-likelihood of output spike counts weighted by kernel 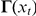 (compare equations 2 and 3). Furthermore, this coding strategy presents three additional advantages. First, it ensures that information about the stimulus can be read out on-line and spike-per-spike, each new spike of a neuron 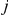 adding a kernel 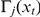. Second, the leak term 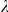 implies that the position inferred from *all* past inputs (i.e. during seconds or minutes of sensory integrations or working memory) can be extracted within a time window of order 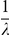 (typically a few tens of milliseconds). This enables both long sensory integration as well as fast computation with leaky devices such as biological neurons. Finally, since the read-out is linear in log probability, combining information from multiple spike trains corresponds simply to using additional read-out kernels. For example, consider another network computing the position of the mouse based on olfactory cues. The total information can be read out by a single decoder applied to the output spike trains of both networks simultaneously. In effect, this performs a product of the two posterior probabilities.

We now derive the dynamics of the output neurons that will ensure that equation (4) holds approximately.

### Network dynamics

#### Inference

In a first step, we need to know what an ideal observer, i.e. an observer that performs optimal inference on the input spikes, would know about the stimulus. We denote it as 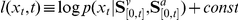 which is the unnormalized log posterior probability of the stimulus given all inputs. Normalization can be neglected since the important information about the stimulus is contained in the shape and location of the distribution.

With the assumptions made in the previous section, we can derive an expression for the ideal observer of the stimulus in the limit of small 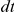:
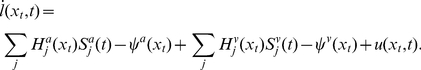
(5)


The ideal observer performs a linear integration of the input spikes weighted by the kernels of their likelihoods. The term 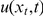 describes the evolution of the log posterior in the absence of input. As a consequence of the drift-diffusion dynamics of the stimulus, 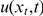 derives from a Fokker-Planck equation and takes the form 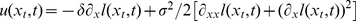 (see Materials and Methods for details).

#### Output generation

Output spike trains shall be generated such that the output read-out, 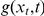, matches the ideal observer 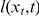. We first discretize the stimulus space and evaluate the posterior at positions 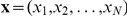, where 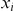 corresponds to the preferred stimulus of output neuron 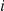. Let us denote 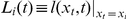 and 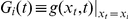. Similarly, we denote 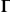 the discretized version of the vector function kernel 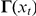, such that 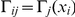.

We propose a spike generation criterion that minimizes the mean squared distance between 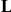 and 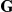. It is schematically illustrated in figure 1C. The effect of a spike of output neuron 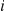 is to add a kernel 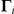 to 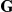. A spike is generated whenever it has the effect of reducing the mean squared distance between 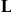 and 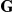, i.e. if
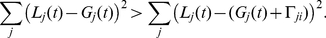
(6)


This criterion ensures that neurons only fire spikes to account for new information about the stimulus that has not previously been reported by their own or their neighbors' activity. Avoiding spike redundancies minimizes the metabolic cost of the code and increases the independence among output spikes.

In contrast to other error measures such as the Kullback-Leibler divergence, the squared distance results in a local integrate-and-fire spike generation rule. Indeed, let us now define the “membrane potential” 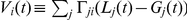, which simply is the difference between input and output log posterior, weighted by output kernel 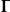. We can show that the temporal evolution of 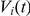 follows leaky integrate-and-fire dynamics (see Materials and Methods for details)
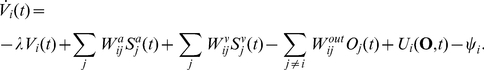
(7)


Output neurons integrate input spikes with feed-forward weights 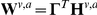 and output spikes with lateral weights 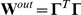, where 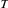 denotes the matrix transpose. The constant bias term 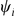 contains information about how informative it is not to receive a spike. Neuron 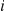 fires a spike if 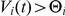, with threshold 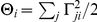. After firing a spike 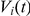 is reset to 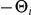.

The slow currents 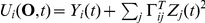 are driven by output spikes and predict the dynamics of the stimulus. Their temporal evolution is given by
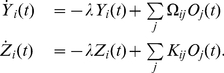
(8)


The weights 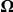 and 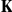 are functions of the output kernel, the leak and the stimulus dynamics: 
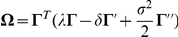
 and 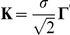, where 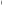 denotes the partial derivative with respect to 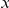.

#### Roles of the different currents

**Figure 2:**
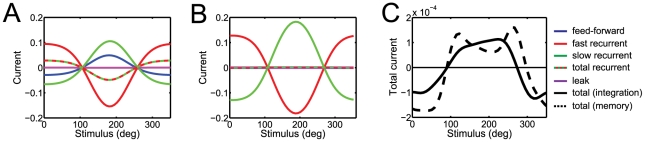
Currents. Averaged currents to a neuron with a preferred stimulus of 180 deg as a function of the presented stimulus location. (A) Currents during the integration period. Feed-forward input currents (blue) are excitatory for stimuli similar to the preferred stimulus of the neuron and inhibitory otherwise. The sum of fast and slow recurrent currents (red-green dashed line) follows an inverted profile of similar magnitude that counteracts the effect of the feed-forward input. The leak current (magenta) is small in magnitude compared to the synaptic currents. (B) Currents during the memory period. Feed-forward inputs are equal to zero. The individual lateral currents are enhanced with respect to the integration period. However, their total sum (red-green dashed line) is balanced and close to zero (see also the black dashed line in C). (C) Total currents (including leak) during the integration period (solid line) and during the memory period (dashed line). In both cases, the contributions of individual currents balance each other out such that the total current is small, slightly excitatory among neurons whose preferred stimuli are similar to the presented stimulus and inhibitory otherwise. The two maxima of the current during the memory period are due to the non-linear component of the slow recurrent currents (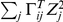) that codes for the stimulus diffusion. It has the effect of broadening the response during the memory period (see figure 3A).

An output neuron receives inputs through fast feed-forward connections (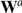 and 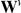), fast recurrent connections (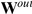) as well as slow recurrent connections (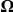 and 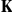). Fast currents are “instantaneous” while slow currents are integrated with the time constant of the decoder 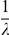. For the sake of simplicity we have assumed that the membrane time constant is the same as the time constant of the decoder. This predicts that fast postsynaptic potentials (PSPs) are exponentials with decay 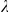 while slow PSPs are Gamma functions (an exponential of decay 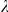 convolved by itself). In practice, the two time constants could differ significantly without affecting performance. In fact, leak currents scale with 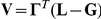 and are in general much smaller than feed-forward and recurrent currents scaling with 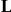 or 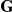. The contributions of leak currents to the network dynamics are therefore negligible (see figure 2). It follows that the membrane potential dynamics could be much faster than the slow currents, as would be the case for instance for 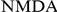 and 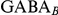 synapses.

Example contributions of the different currents are depicted in figures 2A and 2B. Feed-forward inputs transmit new sensory evidence about a stimulus to the output neurons. Thus, feed-forward currents are globally positive for neurons whose preferred stimuli are similar to the presented stimulus, and negative for neurons whose preferred stimuli are different from it (figure 2A). In contrast, fast recurrent inputs subtract the output population's prediction from this sensory input and hence have opposite signs. Neurons with globally positive feed-forward currents receive negative fast recurrent currents, and vice-versa. Short-range fast inhibition and long-range fast excitation have the effect of avoiding redundancies by only letting one output neuron transmit unaccounted information at a time.

Slow recurrent connections, on the other hand, have two distinct roles. First, they “reintroduce” information that has leaked out, hence making past information available within the time window of integration of the decoder. It is this short-range slow excitation and long-range slow inhibition, mediated by the recurrent connections 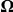 (or more precisely their subpart 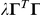), that enables sustained bumps of activity in the output layer and therefore implements working memory. The second role of the slow currents is to take into account the non-stationary dynamics of the stimulus. For example, the stimulus drift is predicted by a spatial derivative of the feed-forward inputs (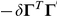, a component of the lateral connections 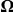), while the stimulus diffusion is predicted by a bimodal current peaking at the position of maximal slope in population response, contributed both by 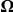 and 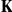. Slow currents hence maintain, shift and widen the global pattern of activity in order to predict the future state of the stimulus.

Altogether, spike generation in our model is deterministic and results from a competition between an integration of evidence from feed-forward and slow lateral inputs, 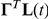, and a prediction from fast lateral connections 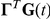. A direct and important consequence of this competition is the maintenance of an almost perfect balance between the global excitatory and inhibitory currents received by each output neuron (figure 2C). Indeed, the total average current is given by 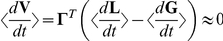, since the network dynamics ensure that 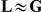. Different choices of kernel 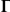 can change the sign of excitatory and inhibitory interactions among output neurons, but total excitation and inhibition is always going to be balanced by the network dynamics. Spikes are caused by unpredictable fluctuations of this total balanced input. Even though output neurons share most of their feed-forward and lateral connections with their neighbors, the resulting output spike trains are asynchronous and have low firing rates (see section on network predictions and discussion).

Finally, we assumed for the sake of simplicity that the same output neuron can both excite and inhibit different target neurons, which is clearly not realistic. A more realistic model can be constructed by using one purely excitatory neuron and another purely inhibitory neuron for each output kernel.

#### Roles of the output kernel 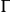 and leak 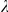


The free parameters of our model are the leak 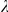 and output kernel 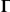. All other parameters are functions of 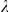, 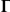, the stimulus dynamics (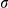 and 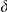) or the input response tuning curves 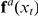 and 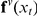 (or, more generally, the input kernels 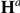 and 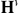).

The kernel 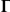 determines the spatial impact or “meaning” of a spike. For example, we can adjust the kernel to give more or less “weight” to each output spike. A larger kernel results in lower activity as less spikes are needed to convey the same information. Thus, if the output kernels are multiplied by a constant 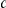, the output firing rates are roughly divided by 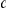. This comes at the cost of fine precision, since changes in log-posterior smaller than the output kernel are not represented.

These output kernels do not necessarily need to be known in advance by the decoder, or any other neural structure extracting information about 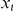 from the output spike trains. They can be estimated (or “learnt”) directly from the tuning curves, 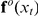, and covariance matrix, 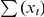, of the output neurons [6]:
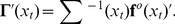
(9)


This relationship holds if the spiking likelihood of the output neurons lies in the exponential family with linear sufficient statistics [6]. We found that decoders using the “true” kernels or kernels estimated using equation (9) were almost identical and performed equally well. Simulation results are reported for the learnt kernel. Equation (9) also shows that the choice of a specific output kernel constrains the tuning curves and covariances of the output neurons.

Similarly, the leak 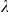 determines the temporal meaning of a spike. It sets the timescale over which information contained in a spike is meaningful. Shorter kernels (i.e. larger leaks) lead to higher firing rates but also more precise tracking of temporal changes in the stimulus. As described in the next section, 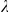 sets the slope of firing rate increase during sensory integration. Additionally, sustained firing rates during working memory are also proportional to 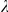.

#### Representation of prior beliefs

Let us briefly go back to our example of the cat and the mouse and say that the cat is looking around to find a mouse to chase. Even in the absence of the mouse, the cat's beliefs on where the mouse is likely to appear is not uniform. The cat might for instance know that there is a family of mice living in a specific bush. It will then base its search mainly on the area around that bush. In other words, the cat has a strong prior belief on where mice are likely to appear.

The prior belief corresponds to the initial value of the log posterior, 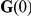, at the onset of the stimulus, i.e. 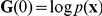. Thus, prior information can be “stored” by applying some external input and driving the output membrane potentials into a specific configuration given by 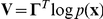 before the start of a trial. The network activity will then maintain this information in memory in the form of a persistent pattern of activity, as it would for a sensory stimulus. Once the stimulus is presented, sensory information will be integrated starting from an initial state determined by this prior.

#### Approximating the nonlocal diffusion term

**Figure 3:**
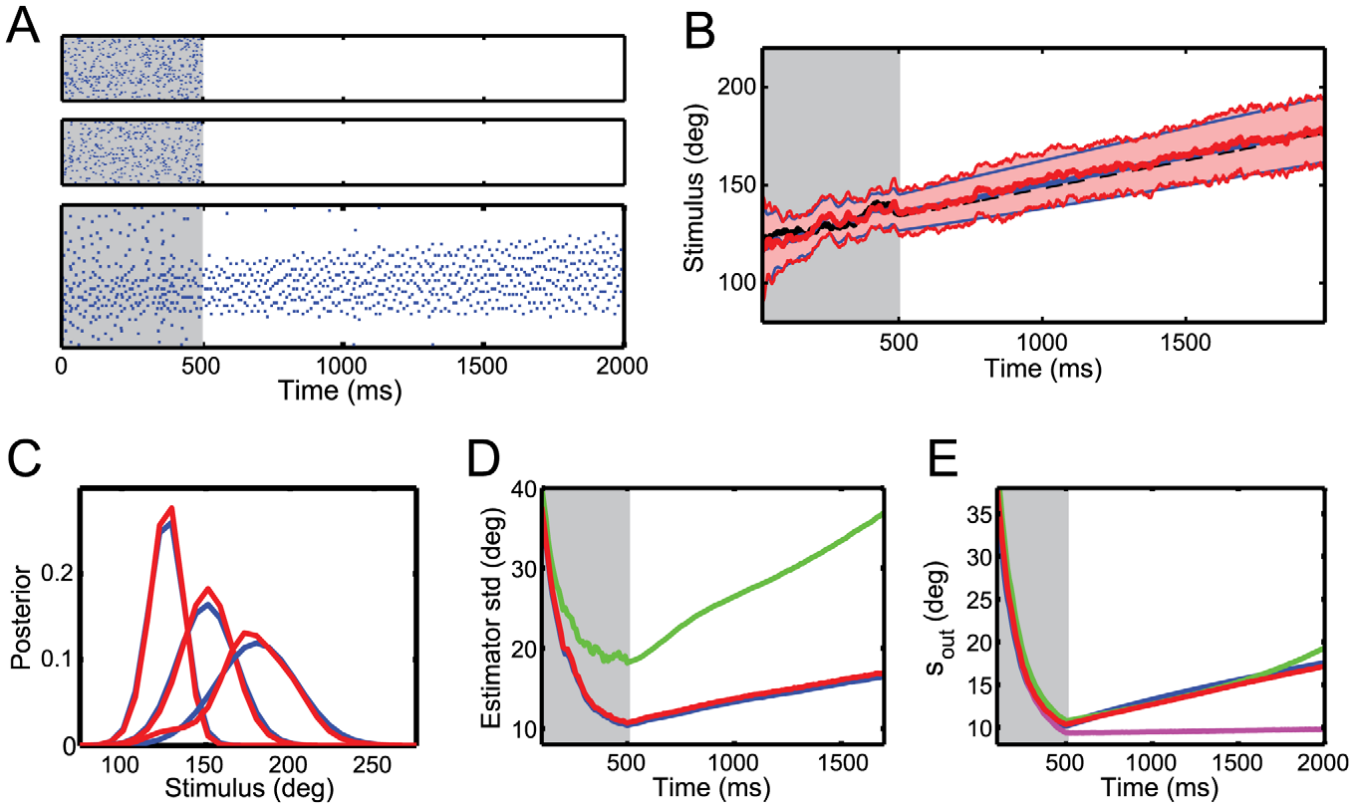
Network performance. (A) Input and output spike trains on a single trial. A stimulus with constant drift and diffusion is presented for 500 ms (gray area). (B) Time evolution of the stimulus posterior for the ideal observer (blue) and the network read-out (red). Thick lines show the mean of the posterior and narrow lines the corresponding width. The stimulus trajectory is shown in black. The dashed black line indicates the predictable (drift) part of the stimulus that the network is tracking during the memory period. (C) Snapshots of the posteriors, from left to right; after 500ms (end of integration period), after 2000 ms and after 5000 ms. (D) Coding performance measured as the standard deviation of the stimulus estimate 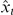 around its real value 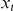. The blue and red curves depict the performance of the ideal observer and the network respectively and the green curve shows the performance of a network without slow currents 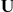. (E) Width of the posterior decoded from the ideal observer (blue), the full network model (described in equations 7 and 8) (red), a network in which we approximate the nonlocal term in the slow currents 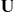 by a linear term (see equation 10) (green) and a network for which we completely remove the nonlocal term (magenta).

If the stimulus includes a diffusive component, the slow current 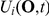 contains a nonlocal and nonlinear term 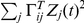. We could imagine that this term is computed by the dendritic trees of the output neurons. It has been shown that dendrites can implement nonlinear functions similar to a two layered neural network [32]. Alternatively, we can approximate the nonlocal term by using the central limit theorem and approximating the posterior by a Gaussian distribution. The slow current 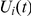 is then given by
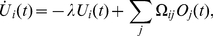
(10)where 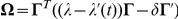. The time varying leak 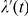 depends on the variance of the posterior distribution, which could be computed with a Kalman filter or directly estimated from the output spike trains. In this paper, we use a simpler approximation and replace 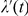 by a constant 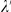, resulting in a fully linear slow current. An example of this approximation will be shown in figure 3E of the next section. All other simulation are done using the full model.

### Model predictions

We illustrate the network dynamics and model predictions using the general task outlined in figure 1A and 1B. Input neurons have bell-shaped tuning curves and generate Poisson spike trains in response to an angular stimulus with constant drift and diffusion. The output neurons follow the leaky integrate-and-fire dynamics of equation (7). The output kernels 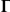 are chosen to be Gaussian shaped. Details of the simulation parameters can be found in the Materials and Methods section. All model predictions described below are largely independent of the specific choices of input and output kernels.

#### Network performance

Figure 3A shows the input and output spike trains on an example trial. A stimulus with constant drift and diffusion is presented for 500 ms during which the output population receives feed-forward sensory input from the auditory and visual layer (top two panels of figure 3A). In the subsequent memory period, input stimulation ceases completely. The output population sustains spiking activity even in the absence of sensory input (bottom panel of 3A). This activity represents a working memory of the stimulus, i.e. a neural correlate of keeping past information available in the time window of integration of output neurons.

The response of the decoder closely matches the performance of an ideal observer (figure 3B and 3C), illustrating the optimality of the model network. This is true for both the decoded posterior 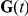 and the distribution of position estimates 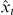 (see methods). During the sensory integration period, the standard deviation of the estimator narrows, reflecting an accumulation of evidence about the stimulus (figure 3D). In the memory period, the sustained spiking activity keeps representing a probability distribution about the stimulus. This posterior tracks the drift of the stimulus, i.e. the predictable component of the stimulus dynamics (figure 3B). The diffusion however is unpredictable and therefore increases the uncertainty about the stimulus. As a result, the standard deviation of the decoded posterior increases over time (figure 3D). However, if we remove the diffusion term (i.e. 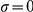), the standard deviation remains constant during the memory period (not shown). In all cases, the standard deviation of the network position estimates remains less than 2% above the standard deviation of an optimal estimator.

Slow currents 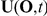 are essential to compensate for the leak in the decoder and predict the drift and diffusion of the stimulus. Without them, sensory integration is suboptimal and information quickly degrades during the memory period (figure 3D). This is a direct consequence of the limited time constant of integration of individual neurons. In fact, neurons lose information at a rate set by the leak 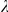. The slow currents compensate for this loss by reintroducing the information that has leaked out and hence making past information available within the time window of integration of a neuron. This turns the neurons into optimal integrators. The nonlinear part of the slow currents can be efficiently approximated by a linear term (equation 10). For an optimal choice of 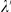, the linearized network performs very closely to the full network (figure 3E).

**Figure 4:**
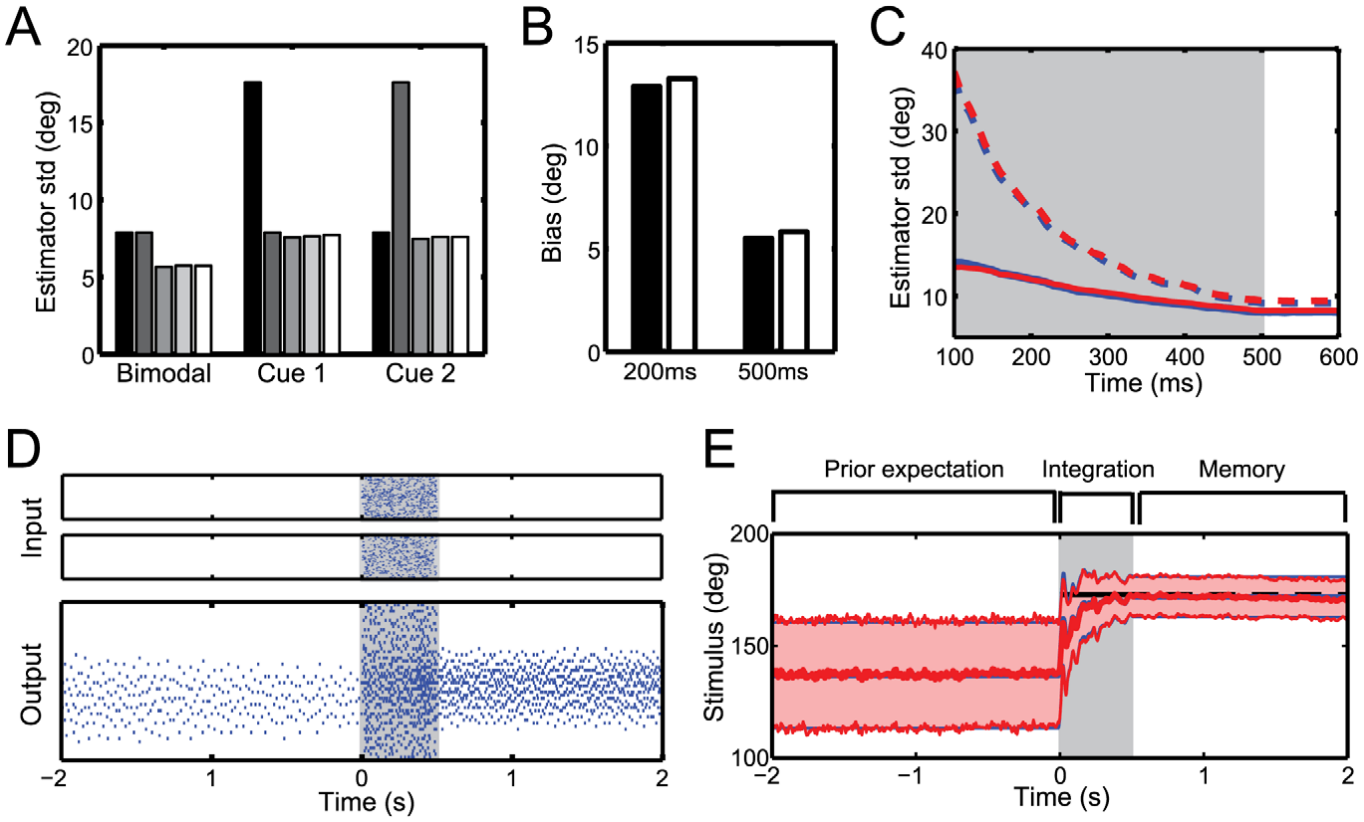
Cue combination and priors. (A) Estimation accuracy for different reliabilities of the input cues: both input cues are equally reliable (bimodal) or one cue is more reliable than the other (cue 1 and cue 2). In each subgroup, bars depict from left to right the encoding accuracy of: cue 1, cue 2, the ideal observer, the network at the end of the integration period and the network after one second in the memory period. (B) Biasing effect of the prior measured as the difference between the real and the estimated stimulus, 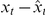. The effect is stronger for short integration times (200 ms, left) than for long integration times (500 ms, right). Black bars show the bias expected for a Bayesian observer, white bars depict the network bias. (C) Standard deviation of the estimator with a Gaussian prior (solid lines) and with a flat prior (dashed lines). A structured prior narrows the width of the posterior. Blue lines denote the ideal observer, red lines the network performance. (D) Input and output spike trains on a single trial. A constant stimulus is presented for 500 ms (gray area). The spontaneous activity before stimulus onset encodes the prior belief about the stimulus. (E) Time evolution of the posterior for the ideal observer (blue) and the network (red). Thick lines show the mean of the posterior and narrow lines the corresponding width. The stimulus is shown in black.

The network implements Bayesian inference and therefore combines visual and auditory cues optimally, weighting each sensory cue according to its accuracy. To illustrate this point, we plot the performance of the network in a bimodal case in which both input cues encode the stimulus with equal accuracy and two “unimodal” cases in which one of the inputs represents the stimulus much more accurately than the other. The accuracy of the sensory input was changed by multiplying the corresponding input tuning curves by a constant 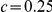. In all three cases, the accuracy of the output estimator, measured by its standard deviation, 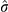, lies within 2% of optimal performance (figure 4A). Thus, the network automatically adjusts to changes in cue reliability.

For the same reason, the network takes prior information into account accurately. Figures 4D and 4E illustrate the spike trains and the decoded posterior distribution on a single trial with a Gaussian prior centered at an orientation of 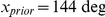. The prior is faithfully represented by the sustained spiking activity before stimulus onset (figure 4D). In this example, a static stimulus is presented to the network for 500 ms. As predicted for an optimal Bayesian observer, the prior biases the position estimates towards 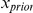 (figure 4B) and narrows the posterior distribution (figure 4C). Moreover, the influence of the prior depends on the reliability of the sensory signal, i.e. the bias is stronger if the stimulus is presented for only 200 ms instead of 500 ms, as shown in figure 4B.

#### Output firing rates

**Figure 5:**
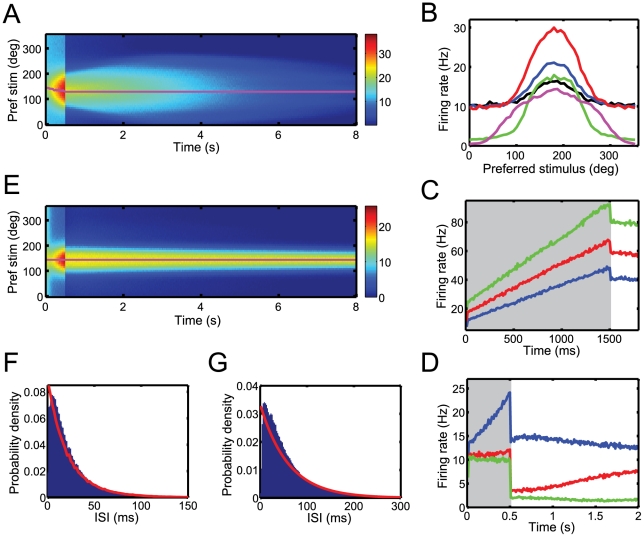
Output firing rates. (A) Post-stimulus time histogram (PSTH) of the output activity in response to a stimulus with constant diffusion. Color indicates firing rates in Hz. The stimulus (magenta line) is presented during the first 500 ms. (B) Tuning curves of a sample neuron. Spikes are counted in 10ms bins centered at 50 ms (black), 200 ms (blue) and 500 ms (red) during the integration period and at 550 ms (green) and 2500 ms (magenta) during the memory period. (C) Traces of the average firing rate of a neuron whose preferred stimulus lies around the peak of the bump of activity. Different curves depict different levels of Fisher information in the input population codes: reference information, 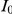 for the regular parameters (red), 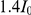 (green) and 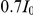 (blue). (D) Traces of the average firing rate of three neurons whose preferred stimuli lie at the peak of the bump of activity (blue), the side of the bump (red) or far away from the bump (green). (E) PSTH of the output activity in response to a static stimulus presented for 500 ms. (F,G) Interspike interval (ISI) histogram during the integration period (F) and during the memory period (G) for a sample neuron. The red line shows the ISI histogram of a Poisson process with the same rate.

The presentation of a stimulus 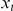 results in a bell-shaped pattern of activity in the output population, peaking at 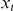. Thus, output neurons are tuned to the position 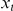 with bell-shaped tuning curves, similarly to the input neurons. However, the shape and amplitude of their tuning curves vary during the entire duration of the trial (figure 5B).

The integration of sensory evidence and its maintenance in working memory is reflected by the instantaneous firing rates of the output neurons. Figure 5A depicts the post-stimulus time histogram (PSTH) of the output neurons in response to a stimulus with constant diffusion. The corresponding tuning curves are illustrated in figure 5B. During the integration period, the firing rates initially jump to a higher level of activity and subsequently ramp up. The gain of the tuning curves increases linearly with time, reflecting an accumulation of sensory evidence. Both the size of the initial response and the slope of the ramping increase in firing rate depends on the accuracy and quality of the sensory inputs. Thus, if we increase the Fisher information available in the input population codes (see methods), firing rates grow faster, reflecting a faster accumulation of evidence (figure 5C). This is reminiscent of neural responses in the parietal cortex during motion integration tasks [27]. The slope of the ramp is also proportional to the leak term 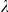. Thus, integrate-and-fire neurons with no leak (or with time constants significantly longer than the effective time constant of the dynamic stimulus) would have constant firing rates during sensory integration. This is predictable since 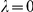 implies that the decoder is able to integrate output spike trains over the entire duration of the trial. It is therefore not necessary to represent accumulated sensory evidence on-line. In all cases, neural activities eventually saturate at a constant level, since the diffusive noise limits the precision with which the stimulus can be encoded (not shown here).

Firing rates during the memory period have a lower baseline activity but similar tuning as during the integration period. Over time, tuning curves and population activity decrease, broaden and eventually disappear (figure 5A and 5B). As a result, the instantaneous firing rates during the memory period are not constant but vary dynamically, ramping either up or down. Figure 5D shows the average firing rates of three neurons whose preferred stimuli are located around the peak of the persistent bump of activity (blue), the side of the bump (red) and far from the bump (green). Similar neural behavior has been observed in parietal and prefrontal brain areas during working memory tasks [27], [33], [28]. Our model suggests that such ramping behavior might reflect the widening of the posterior over time due to an accumulation of uncertainty about the represented variable. Thus, ramp-like changes in firing rates during working memory tasks could be a signature of a gradual decrease in confidence for this memory.

However, the behavior of the network is different in the absence of diffusion. The network is then able to maintain information about the stimulus over very long timescales, reflected by a neutrally stable bump of activity (figure 5E). The firing rates during the memory period are thus constant over time for a static stimulus. However, the amplitude of the sustained bump of activity depends on the amount of accumulated sensory evidence (figure 5C) as well as on the neural integration time constant. Indeed, the sustained firing rates necessary to maintain a constant log posterior, 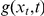, are proportional to 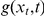 multiplied by the leak 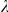 (see equation 3). Thus, persistent activity is larger for more informative sensory inputs or stronger leaks. Notice that neurons and decoders without a leak would not exhibit any sustained activity.

This implies that our working memory model differs from previous models that are based on line attractor dynamics [31], [30]. For these bump attractors, neural dynamics settle onto stereotyped activity profiles whose peak positions encode the most likely stimulus values. The probabilistic information associated with these values, however, is lost. In contrast, our network acts as an optimal integrator that maintains the sensory information it has received in the past. Consequently, various patterns of activity that differ in shape and amplitude can be sustained.

**Figure 6:**
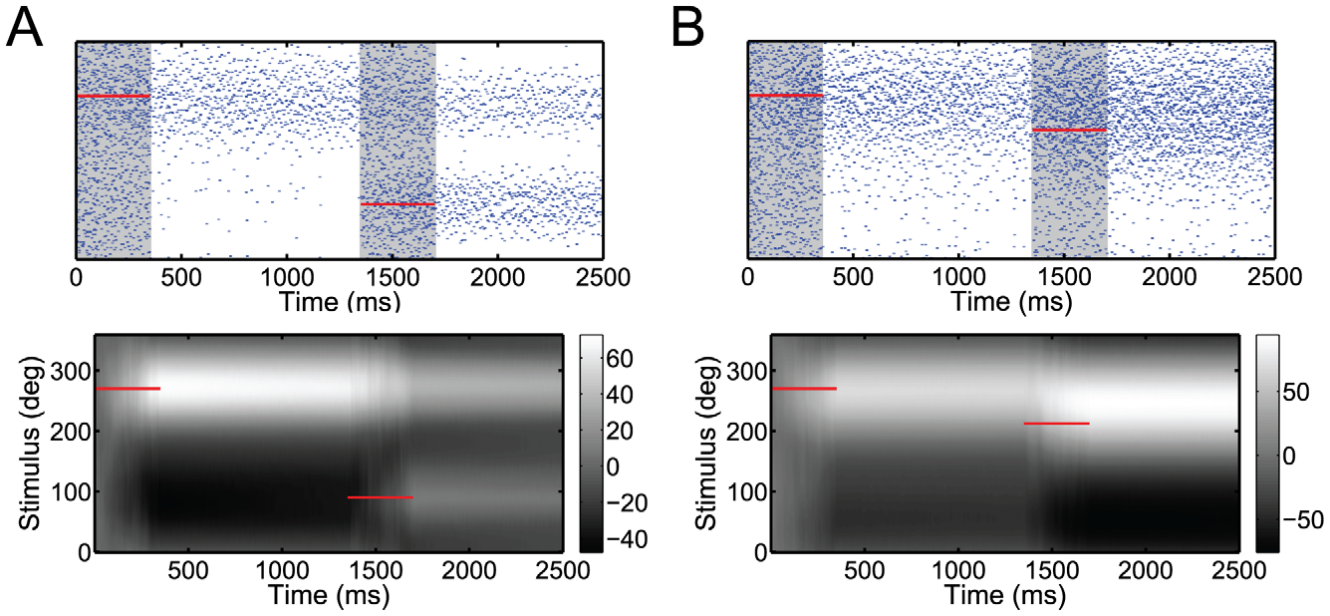
Response to multiple stimuli. Two static stimuli (red lines) are consecutively presented to the network for 350 ms each. They are separated by a delay time interval of one second. Their spatial distance is (A) 180 deg, and (B) 45 deg. Top row: Spike trains on a single trial. Bottom row: Time evolution of the unnormalized log posterior (gray scale representation). The simulated network contains 200 instead of 50 neurons for better visual clarity.

In particular, our network can maintain multi-modal posterior distributions reflected in multi-modal patterns of activity. Figure 6 depicts a case in which two different stimuli are consecutively presented to the network with a delay interval of one second. Both stimuli are presented for equal time periods of 350 ms. Their representation depends on the relative distance between them. If the stimuli are presented far away from each other, the network sustains two spatially distinct bumps of activity (figure 6A). Both stimuli are also clearly represented in a bimodal log posterior distribution. However, if the two stimuli lay close together, individual bumps fuse into a single bump (figure 6B). As a consequence, the log posterior becomes unimodal, peaking in between the two stimuli. Thus, the accuracy at which information about individual stimuli can be resolved is limited by their spatial discrepancy.

#### Output spike train statistics

The resulting output spike trains are asynchronous and spike times are not reproducible from trial to trial. They exhibit properties very similar to Poisson processes. Thus, the interspike interval (ISI) distributions of the output spike trains are quasi-exponential in both integration and memory period (figure 5F and 5G). We find coefficients of variation (CV) of 0.97 in the integration and 1.06 in the memory period. Fano factors are about 1.4 in both periods. We also observe only small cross correlations between different neurons. Correlation coefficients never exceeded 0.001.

The sensory stage in our model is noisy, reflected by the Poisson firing of the input neurons. In contrast, output neurons generate spikes deterministically. Despite this fact, their spike trains resemble independent Poisson processes. This is true even during the memory period when the network activity is self-sustained and no noise is introduced by the external inputs. This eliminates the possibility that the output statistics are directly inherited from the Poisson distributed, feed-forward inputs and raises the question of where this variability comes from. In particular, can the responses of the network be considered to obey the predictions of a rate model?

We hence investigate the origin and role of this variability by using two approaches: A perturbation approach to study the dependency of output spike trains on initial conditions; and a decoding approach where we study how well the spike train of an output neuron can be predicted from the activity of the other neurons in the population.

**Figure 7:**
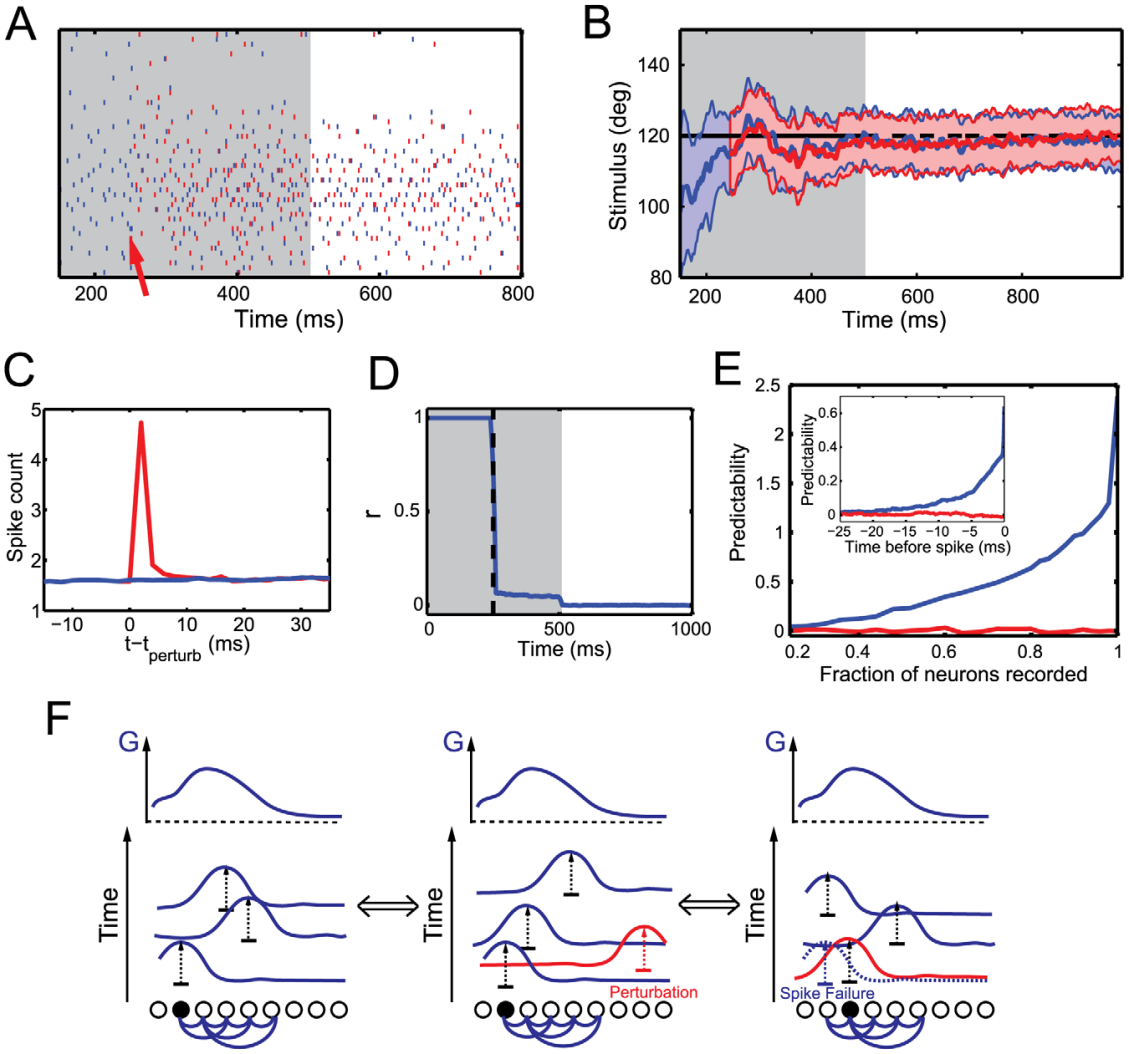
Spike train variability. (A) Output spike trains for two runs (blue and red) of activity starting with the same initial conditions. The red run is perturbed by the injection of one extra spike (shown by the red arrow). (B) Time course of the posterior of the two runs. (C) PSTH of the control (blue) and the perturbed (red) runs. The extra spike is injected at 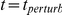. Spikes are counted in 2 ms time bins and averaged over all neurons and over 10000 trials. (D) Time course of the normalized cross-correlation between the two runs of activity. The vertical dotted line indicates the time at which the perturbation (one extra spike) was added. (E) Predictability (equation 33) of the activity of an output neuron if we record from a fraction 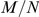 neurons of the output population. The predictability for neuron 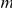 is plotted for spikes that are generated from the deterministic network (blue) or from a Poisson process (red). The rightmost predictability (at a fraction of 1) corresponds to the predictability of the measured, i.e. not predicted, membrane potential. The inset shows the increase in predictability previous to a spike (for a fraction of recorded neurons of 0.8). (F) Schematic illustration of the error correcting properties of the network. The left panel shows a reference spike train. Each spike adds a kernel that when added together give the log posterior G (top). If an extra spike is added (middle panel, red spike), the spike train is reshuffled in a way that keeps the total log posterior constant. If the initial spike fails to be elicited (right panel, blue dotted spike), a neighboring neuron recognizes the “hole” of information transmission and fires a spike to fill it. This changes the initial condition (first firing neuron in black) and therefore shuffles the spike train. The total log posterior remains the same.

*Perturbation approach*. We consider the smallest possible perturbation; one additional output spike. The injection of only one extra spike disrupts the spike pattern and reshuffles the times of all subsequent spikes in the population (figure 7A). This effect is observed regardless of whether the extra spike is injected during the memory period or during the integration period. The average firing rates of the output neurons sharply increase directly after the perturbation, indicating that each extra spike produces many other extra spikes in its postsynaptic targets (figure 7C). This rise in firing rate quickly decays, such that the perturbed and unperturbed firing rates become indistinguishable within 10 ms after the injection of the extra spike. Such short-lived increase in population firing rate due to an added spike has recently been reported *in vivo* based on stimulation and recordings in rat barrel cortex [34].

Figure 7D shows the time course of the normalized cross-correlation between the perturbed and unperturbed spike trains. The addition of an extra spike induces a fast drop of this correlation. This is characteristic of a chaotic network [35], [36] in which two initially identical trajectories quickly diverge after a small perturbation.

The encoding properties of the output neurons are thereby not affected. The decoded posterior still matches the ideal observer closely (figure 7B). This shows that there is a multitude of spike patterns that can optimally encode the same information. Which pattern is chosen by the network strongly depends on initial conditions and small perturbations (see the schematic illustration in figure 7F).

We observed the same characteristics if a single output spike fails to be fired. Spike patterns are again completely reshuffled while coding performance is unaffected. Moreover, our model is robust to even frequent spike generation failure. The reason lies in the error correcting property of the code. If a spike generation fails it is compensated by a spike from another neuron that adds a similar kernel to the posterior, as illustrated in figure 7F.

*Decoding approach*. We apply a decoding analysis during the memory period, in which the network relies only on its deterministic internal dynamics, and we consider a static stimulus without drift or diffusion.

Let us first assume that we record from the entire population of output neurons. We want to know how well the spike times of a single neuron 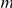 can be predicted by the responses of the 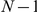 other output neurons. Notice that if the spike trains were independent Poisson processes and hence completely uncorrelated, the spike times of neuron 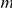 could not be predicted at all. In contrast, in our network, the membrane potential of neuron 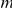 depends on the spikes from the 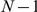 other neurons.

We can predict the spike times of neuron 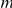 by estimating when its membrane potential (equation 7) will cross the firing threshold. This prediction will not be perfect since the initial state of the network 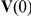 is unknown. However, we can still predict spike times with millisecond accuracy with such a method.

Let us now suppose that we record (more realistically) from a subpopulation of 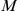 output neurons. The responses of the 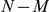 other neurons in the full population is unknown. We want to know how well the spike times of recorded neuron 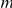 can still be predicted by the responses of the 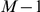 other recorded neurons. Our strategy is to treat the 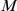 recorded neurons as if they represented the whole output population, using their spike trains to predict the membrane potential of neuron 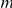, 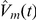 (see methods). In this case, the spike times cannot be predicted with millisecond accuracy anymore. However, 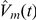 is still correlated with the true membrane potential, and it increases shortly before an actual spike in neuron 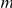 (figure 7E, inset). We measured “predictability” by how significant this increase in predicted membrane potential is at the time of a spike (see methods). The predictability of an uncorrelated Poisson spike train would be zero.

As shown in figure 7E, the predictability is high when most of the population is taken into account. However, predictability decreases with the portion of output neurons that are recorded simultaneously. It becomes indistinguishable from a rate code with Poisson distributed, uncorrelated spike trains if less than 25% of the neurons in the population are recorded. In cases where it is possible to record from a large subpopulation, this analysis provides a specific, experimentally testable prediction.

#### Robustness

We have previously seen that our network is robust to small perturbations and spike generation failure. We are now going to show that it is also robust to synaptic noise. Synaptic background noise is a prominent source of neural noise [37]. Cortical neurons receive barrages of inputs that are largely uncorrelated with feed-forward stimuli [38] and this noisy input is sufficient to affect the spiking properties of these neurons [39]. We model synaptic background noise as an additive white Gaussian noise term on the membrane potential of the output neurons. This noise current has a mean strength of zero and a standard deviation of 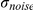. It increases the standard deviation of the total input that output neurons receive (including feed-forward and recurrent inputs) while letting the mean input unaffected. This results in a decrease of the signal-to-noise ratio of the total input, SNR = mean(input)/std(input), measured as the ratio of mean input to the standard deviation of the input. Thus, synaptic noise introduces additional uncertainty about the stimulus.

**Figure 8:**
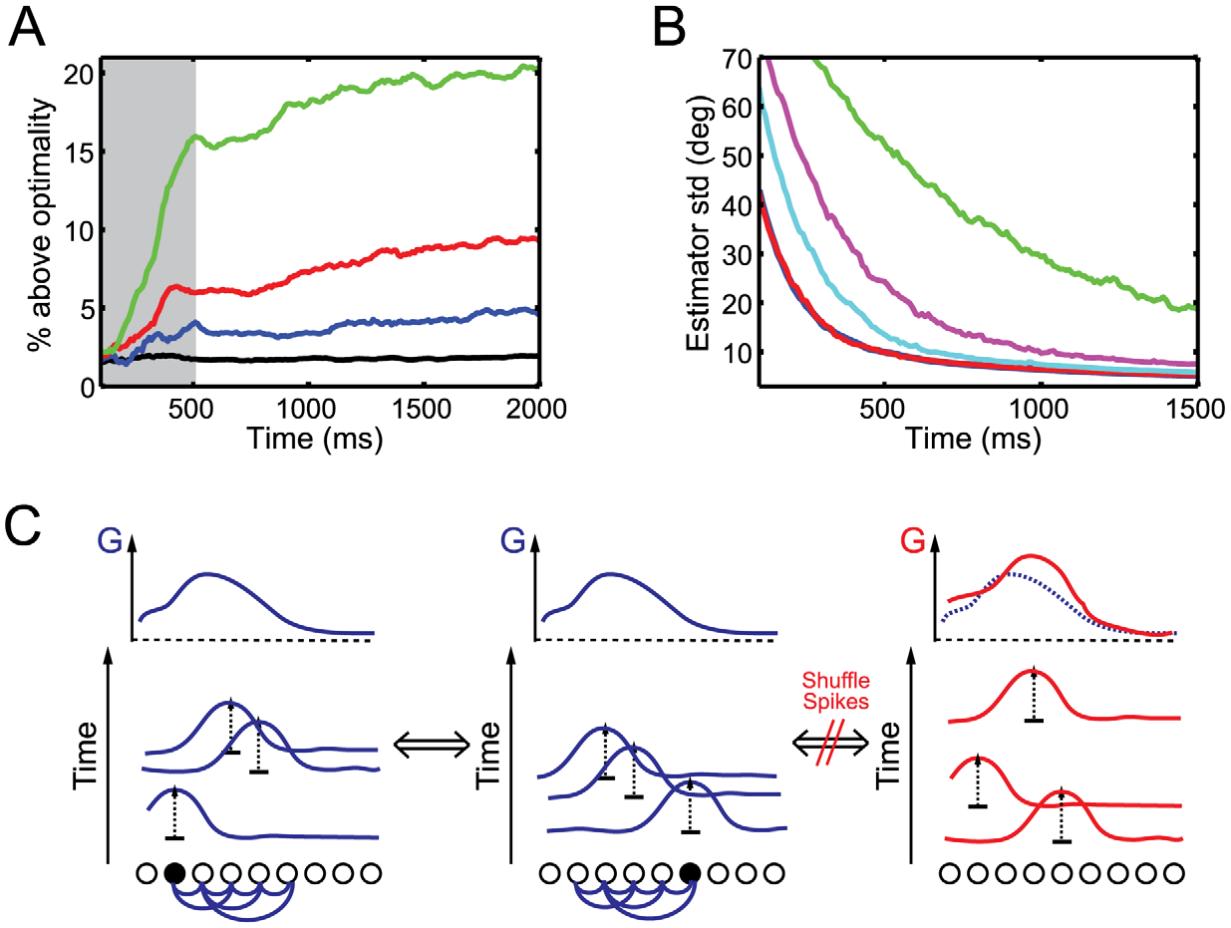
Robustness to noise. (A) Coding performance of the network in the presence of synaptic background noise. The vertical axis plots the percentage excess of the standard deviation of the stimulus estimator above its optimal value. Results are reported for percentual decreases in the signal-to-noise ratio, SNR = mean(input)/std(input), of 0% (black), 20% (blue), 50% (red) and 100% (green). A static stimulus is presented during the first 500 ms (grey area). (B) Coding performance of a stochastic network for different output gains: 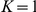 (green), 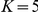 (magenta) and 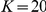 (cyan). The ideal observer is plotted in blue and the performance of the deterministic network in red. A static stimulus is presented during the entire 1500 ms. (C) Schematic illustration of the difference between deterministic and stochastic spike generation. The left and middle panel show two spike trains encoding the same information but starting from different initial conditions. However, neurons in the output population are recurrently connected and “know” therefore perfectly well, when to fire a spike such that the log posterior 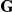 is represented. If the lateral connections are removed, neurons fire stochastic spike trains that look similar to the deterministic ones but do not encode the same log posterior.

Figure 8A shows the effect of different strengths of synaptic noise on the network. With increasing noise strength, the standard deviation of the stimulus estimator lies increasingly above its optimal value. However, even at a noise level that reduces the signal-to-noise ratio by 100%, the network performance at the end of the 500 ms integration period is only 15% worse than optimality. A SNR reduction of 20% only slightly affects the network performance. In the memory period, network performance decreases further although more slowly. This indicates that the network is most sensitive to noise at an early stage of the integration period. Once the stimulus posterior has sharpened, the network is more robust to noise perturbations. Altogether, our model is robust to even high levels of synaptic background noise.

This robustness to even large levels of synaptic noise is another consequence of the error-correcting property of the code. Synaptic noise will lead neurons to reach their firing threshold even if their kernel does not decrease the mean squared distance between 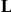 and 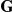 (see figure 1C). However, other output neurons will detect this temporary increase in prediction error in their membrane potential and fire spikes to compensate for it.

For a similar reason, our network is robust to changes in the connection strengths between neurons. Scaling all recurrent synapses by 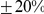 from their optimal values leaves the network performance largely unaffected (figure not shown). This contrasts with networks based on line attractor dynamics (e.g. [40]), which require connections to be tuned with better than 1% accuracy (see however [41]).

#### Comparison to a rate model

Despite its deterministic nature, our model exhibits firing statistics comparable to a rate model with independent Poisson noise for which spike times do not carry information. Thus, the question arises whether we could implement the same computations equally efficiently with stochastically generated spikes? In particular, if we consider biological networks with thousands of neurons, averaging responses from large populations of neurons might render the contribution of each spike unimportant. In this case, spike-based and rate-based approaches might become equivalent. In the following, we show that this is not the case. A deterministic spike generation rule is crucial for efficient information transfer even in very large networks.

To show this, we started by implementing a version of the probabilistic population code of Ma et al. [6]. These authors have shown that optimal integration of information from two population codes reduces to a linear combination of their neural activities. In the context of temporal sensory integration, the predicted output firing rates, 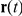, correspond to the cumulative spike counts [7]. Thus, the output firing rates are given by
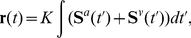
(11)where 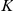 represents the gain of the output neurons. As in our model, the output rates 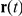 represent the stimulus posterior distribution optimally and on-line. In particular, activities increase linearly over time to account for the accumulation of sensory evidence. In order to avoid saturations of neural activities, Beck et al. [7] proposed a form of on-line normalization, effectively using a time varying gain 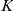. This does not change the main conclusion of this section. For the sake of simplicity, we consider 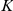 to be constant.

We now examine the consequence of firing spikes stochastically with rate 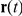 rather than representing this accumulated evidence deterministically. We measured the performance of the stochastic network with the on-line decoder described in equation (3) and using the optimal output kernels 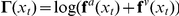.

Figure 8B depicts the performance of the stochastic network for different values of 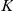. The stochastic network behaves qualitatively like an ideal observer, i.e. it accumulates evidence and its error decreases over time. Moreover, for large gains 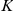 and long integration times, the performance of the stochastic network approaches the performance of an ideal observer of the sensory input (i.e. about 10% above optimality for 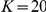). However, for shorter sensory integration periods (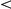
500 ms), the performance is poor even for large gains. Moreover, the output gain 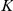 has to be much larger than one. This implies that the stochastic network requires many more output spikes than input spikes (about 15 times more in this example) in order to avoid destructive information losses between the input layers and the output layer. By contrast, our network fires half as many output spikes than input spikes. We found that we could even lower that amount to 5 times less spikes in the output layer than in the input layers by increasing the size of the output kernels without any significant degradation in network performance.

A neural system clearly cannot afford to spend 15 times more resources at each processing stage. Moreover, this cost of stochastic spike generation does not decrease with the size of the input and output neural populations. In the limit of large numbers of neurons/spikes, the variance of the stochastic network estimate approaches 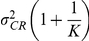, where the Cramer-Rao bound 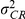 is the variance of an optimal estimator (see methods). Efficient information transfer can only be achieved at the cost of large values of 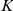, i.e. many more output spikes than input spikes.

## Discussion

In this article, we have revisited population coding with spiking neurons in the context of dynamic stimuli. Starting from first principles, we have demonstrated that networks of laterally coupled integrate-and-fire neurons can integrate and combine sensory information about a dynamic stimulus in close approximation to an ideal observer. In the absence of sensory input, these networks either represent the stimulus prior probability in their spontaneous activity before stimulus onset or they represent a working memory of the inferred stimulus posterior in their sustained activity after integration. These memories thereby keep tracking the underlying stimulus dynamics.

An important innovation of our model is that it encodes working memories representing an entire stimulus distribution rather than only a single stimulus value. It thereby distinguishes itself from other working memory models in the literature. Most working memory models are bi-stable attractor models [31], [30] in which the sustained activity settles to a stable pattern independently of integration time or stimulus contrast. It is clear that such a stereotyped activity profile can only code for the most likely stimulus. Information about the uncertainty associated with the stimulus is lost. In contrast, our model is not based on bi-stability or line attractor dynamics but on an integration of past sensory evidence. In the presence of diffusion (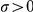), the only stable state is the quiescent state, which corresponds to a flat probability distribution. In the absence of diffusion, the network maintains any pattern of activity that is evoked by past sensory stimulation. However, sensory stimuli in the real world are never “truly” stable. Moreover, any form of stochasticity in neural processing will result in a slow but constant accumulation of errors (see for instance the progressive decrease in performance due to synaptic background noise in figure 8A). Both of these properties will lead to working memories that are not completely stable, but eventually relax towards a quiescent state, i.e. a flat posterior distribution. In agreement with this prediction, the precision of a working memory for static stimuli degrades with the duration of the delay [42].

We propose that cortical neurons are primarily predictive encoders rather than stochastic spike generators. Integrate-and-fire dynamics as well as a competition between neurons only allows the generation of spikes that contain new information about the stimulus, i.e. information that has not yet been signaled by the neural population. Each spike therefore carries a precise meaning. As a consequence of the above mentioned properties, small networks of only tens of neurons can encode stable memories. Persistent, asynchronous memory states are notoriously difficult to achieve with small networks of integrate-and-fire neurons. Our model on the other hand is largely free from laborious fine tuning. It provides a functional interpretation of parameters such as lateral connections and synaptic dynamics, and could be used as a guideline to find optimal parameters in biophysically plausible networks. For instance, the slow currents 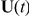 in our framework might be mediated by a combination of slow excitatory NMDA synapses and slow inhibitory 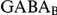 synapses. NMDA synapses have been identified by previous studies as a potential requirement for robust working memory responses [30], [43], [40].

In our framework, prior beliefs correspond to setting the network into an initial state 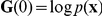. As an example, we proposed an implementation of a sustained pattern of baseline activity, equivalent to a working memory for an input provided before the start of the trial. Similar mechanisms for implementing priors using external inputs have been suggested in other theoretical studies [6]. This would predict that baseline firing rates are modulated by prior assumptions of a subject, for example by stimuli experienced in the recent past. However, “long-term” prior beliefs could also be implemented by the choice of output kernels. Thus, the density of preferred stimuli in the neural population could be chosen non-uniformly and such that 
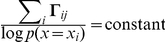
 [44]. In this case, the prior would be represented by all neurons firing at a constant, low baseline firing rate. This predicts no structure in the baseline response prior to stimulus presentation, and no direct influence of the prior on the tuning curves of individual neurons. In support of such a mechanism, perceptual learning causes an increase in neural representation for more frequently experienced stimuli [45], [46], [47].

Another important aspect of our approach concerns its interpretation of neural variability. Traditional population coding approaches clearly separate “signal”, encoded in rate modulation, and “noise”, encoded in the spike count variance. Rate models, such as linear-nonlinear Poisson (LNP) neurons [48], rely on stochastic spike generation for generating realistic spike trains. Individual spike times do not carry any meaning while spike train variability is interpreted as noise. A problem arises when such rate units are used to perform sensory integration. In this case, while output units can compensate for the neural noise by integrating information over cues and time, they “throw away” part of this information by firing spikes stochastically. Thus, Lochmann et al. [49] have shown previously that stochastic firing strongly degrades the information transfer capacity of single neurons that represent a time varying binary stimulus. Here we show that this is also the case for continuous stimuli, except if the neural system is willing to largely increase the amount of resources (i.e. spikes, neurotransmitters) it devotes to each sensory variable.

Our approach provides an alternative account for the origin of neural variability observed in cortical networks. Stochastic firing is not a good description of noise in single neurons [50], [51]. Instead, it has been proposed that this variability originates in chaotic dynamics of recurrent networks of integrate-and-fire neurons with balanced excitation and inhibition [35], [36], [52]. This perfectly agrees with our findings since our network shows characteristics of a chaotic system in the absence of sensory input. However, we show that these dynamics cannot be equated to noise. They only reflect the fact that multiple deterministic trajectories (i.e. spike patterns) encode the same information (figure 7). Albeit chaotic, this network can conserve and transmit information perfectly. At the same time, the network is self-correcting and robust to types of noise that have been reported in cortical neurons, such as spike generation noise or synaptic noise [37],[38].

It might appear paradoxical to assume input neurons corrupted by Poisson noise while using perfectly deterministic output neurons. However, input noise in our model is meant to represent unavoidable sources of sensory noise, such as the stochasticity of our sensors in the first signal transduction stages (e.g. thermodynamical/quantum mechanical noise in the photoreceptors). This initial noise sets a bound on how much information is available for further processing stages. We used population codes with independent Poisson noise as inputs for the sake of convenience and because such variability is expected as a consequence of predictive coding. However, the same networks can process any noisy inputs whose log-likelihoods can be computed on-line. Our preliminary findings suggest indeed that our model can construct population codes with Poisson-like firing statistics for almost any type of noisy sensory input, including input that is not Poisson, not spiking or not a population code. Consequently, Poisson distributed input in our model does not represent noise in the input neurons but the outcome of previous optimal neural processing of the sensory input.

Our hypothesis can be tested experimentally in cases where one is able to record simultaneously from a significant portion of the population. Since our model assumes a strong level of inter-connectivity and shared input, a population could correspond to a local, relatively small network such as a micro-column, rather than a large and diffuse network containing millions of neurons. Our model predicts that the larger the simultaneously recorded population, the better one can predict individual spike times, using methods described in section “Output spike train statistics”. On the behavioral level, our model predicts that humans should be able to memorize entire probability distributions. This could be tested by a simple cue combination experiment, in which two cues about a stimulus (e.g. a visual and an auditory cue about the location of an object) are presented with a temporal delay. If subjects keep track of the uncertainty associated with the first cue, they should still behave like optimal Bayesian observers when combining information from the two cues after the delay period.

We are not the first authors to propose a spiking network for optimal cue combination and sensory integration. Ma et al. [6] implemented probabilistic population codes for cue combination, and more recently for temporal integration of evidence in a motion integration tasks [7] with either conductance-based integrate-and-fire neurons or stochastic LNP neurons. However, their theoretical approach is based on firing rates, and the simulated spiking networks are used to show that the sums of spike counts predicted by an ideal observer can also be implemented by spiking neurons. The authors show that the output layer behaves as an ideal observer when comparing uni-modal with bimodal cue combination or when observing how quickly information accumulates over time. However, they concentrate solely on the information contained in the output layer for the different conditions: unimodal versus bimodal or high versus low levels of sensory noise. They do not measure the performance of the spiking network in terms of how much information is conserved or lost in the transfer from input to output spike trains. Our results suggest that while their approach is indeed optimal if outputs are analog firing rates, it becomes suboptimal when translated into noisy spike trains (except if there are many more output spikes than input spikes). In contrast, our model can be used to implement a probabilistic population coding framework directly with spikes rather than with rates.

Other authors have considered log probability codes [9], [53], [8]. For example, Rao [53] proposed a network of integrate-and-fire neurons performing approximate Bayesian inference. Similar to our model, the membrane potentials were interpreted as log posteriors. However, this model encoded posterior probabilities in terms of instantaneous firing rates rather than considering spikes as deterministic prediction errors.

Our approach is similar to the “spiking Boltzmann machine” proposed by Hinton and Brown [21]. This model, however, performed approximate and not exact inference, and did not provide an explicit, local spike generation rule. Another related approach, termed fast population coding (FPC) [23], [18], was applied to more general stimulus dynamics described by Gaussian processes. This model is particularly relevant for very sparse input (few input spikes) and functions by adding more output spikes, hence rendering linear decoding easier. However its spike generation rule (using KL divergence) is non-local, requiring supervised learning of the lateral connections in order to approximate it. In contrast, our model works with a local spike generation rule, essentially compressing the code, but is optimal only for Markovian dynamics.

We assumed that output neurons “know” the parameters of the input noise and stimulus dynamics. Sensory noise, stimulus drift and diffusion are hard-wired in the weights of feed-forward and lateral connections. For the sake of simplicity, we considered simple stimulus dynamics with a constant drift 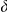 and diffusion 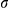. However, our approach can be extended in a straightforward way to state dependent drifts 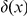 and diffusions 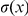. We have seen that the input and output kernels can be learnt from the input and output tuning curves and covariance matrices. Thus, “slow” lateral connections predicting drifts and diffusions could be learnt using Hebbian-learning rules. However, a given network is designed for a specific set of stimulus parameters. Ideally, we would want output neurons to estimate these parameters online during the presentation of a stimulus, for example if the stimulus speed changes suddenly. This could be implemented by multi-dimensional networks representing dynamical parameters [54]. Thus, the state variable 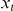 could contain additional dimensions for velocity, acceleration, force, etc. The capacity of such networks to track their stimulus would only be limited by combinatorial explosions as more stimulus dimensions need to be represented.

## Materials and Methods

### Ideal observer

Here we derive an expression for the ideal observer of the log posterior 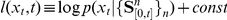, where 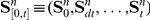 denotes the spike trains of the input neurons in population 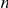 in response to dynamic stimulus 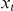. The ideal observer integrates the inputs from 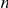 populations that represent 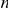 different cues about 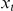.

The total response 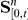 can be divided into the response at the current time step 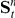 and the response history 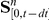. The population response at time 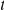 is a binary vector 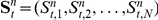 where 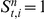 if an input neuron 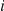 fired a spike at time t and 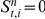 otherwise.

We can use Bayes' rule to write the conditional probability of the stimulus given the past history of activity patterns,
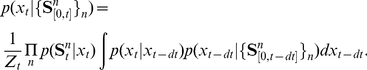
(12)


This equation expresses the current posterior stimulus probability as a spatially averaged version of the past stimulus probability, weighted by the current response probabilities and properly normalized by 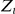. We have assumed that the response likelihoods are independent among input populations and only depend on the current stimulus location. We can turn the multiplications in equation (12) into sums by passing to the log domain,
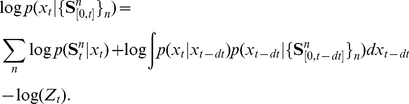
(13)


The normalization term, 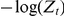, corresponds to an additive constant that does not change the shape and therefore the information content of the log posterior. We will therefore neglect this term in what follows.

The response likelihood 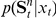 is assumed to belong to the exponential family with linear sufficient statistics, i.e. the firing probability of a neuron in a small time window 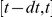 can be written as 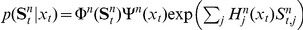, where 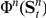 and 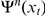 are arbitrary functions and 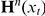 is a kernel that is related to the neurons' tuning curves 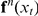 and their spike count covariance matrix 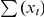 through the relation [6]

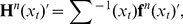
(14)where 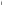 denotes the derivative with respect to 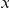. We can then write the likelihood in its log form
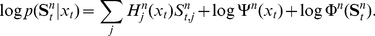
(15)Equation (15) takes a particularly easy form if we consider independent Poisson processes. In this case we find that the kernel 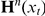 is linked to the logarithm of the tuning curves 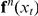 by 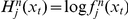 and a bias term is given by the sum of tuning curves 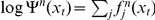. The term 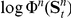 acts as a normalization term and is neglected.

Let us now move to the term 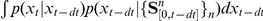. The factor 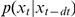 represents the probability that the stimulus moves from 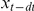 to 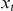 in the small time interval dt. This probability is independent of the starting position, such that 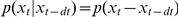. This turns the term of interest into a convolution that we can expand and express as
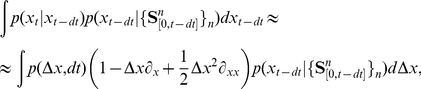
(16)where 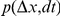 denotes the probability that the stimulus moves by 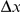 in time interval 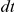. Since 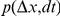 is a probability density 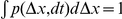. If we assume the stimulus to follow the drift-diffusion dynamics from equation (1), 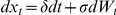, where 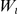 is a Wiener process, we can express the remaining sums in equation (16) as 
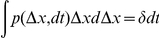
 and 
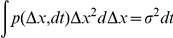
. Using these identities together with equation (16) and taking the log we find
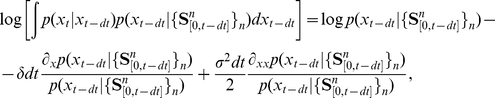
(17)where we have Taylor expanded the log to first order. It can easily be verified that
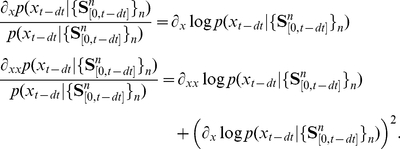
(18)We can use these identities and combine equations (13), (15) and (17) to find the temporal evolution of 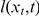 in the continuous limit 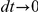:
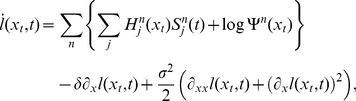
(19)where 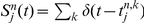 denotes input spike trains with 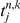 the k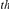 spike of neuron 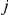 in population 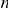.

### Neural approximation to the ideal observer

Here we derive an approximation to the ideal observer that is implemented by the leaky integrate-and-fire neurons in the output population described in equations (7) and (8) of the main text.

We first introduce a discretization of the stimulus space given by 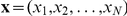, where 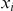 corresponds to the preferred stimulus of neuron 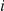. Each neuron therefore codes for the value of the log posterior distribution at its preferred stimulus, which we denote 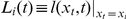. We want the output spike trains to encode a distribution 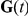 that closely approximates 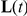, i.e. 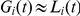 for all 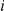. Additionally, following equation (3) the dynamics of 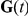 are given as
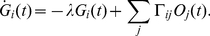
(20)

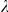
 denotes a positive leak term and 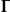 is a freely chosen weighting kernel.

When inferring the input log posterior, 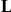, in a neural system, one cannot simply use equation (19) because individual neurons do not have direct access to the spatial derivatives of 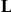. However, if we choose a spike generation mechanism which ensures that 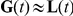 at all times, we can use the recurrent spikes to approximate the spatial derivatives of 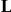 and rewrite equation (19) in a discretized form as
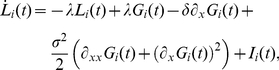
(21)where 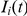 denotes the input to neuron 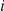 at time 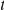. Notice that we have introduced a linear leak 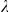 in 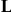 and compensated for it by adding a corresponding fraction of 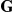.

We now define 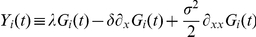. To find the time evolution of 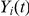 we need to calculate the time derivative of the spatial derivatives of 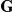. Using equation (20) we get
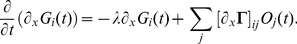
(22)


A similar equation is found for the second spatial derivative of 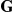. Combining these equations with the definition of 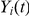 and denoting the spatial derivative with respect to 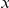 by 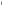 we get
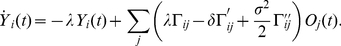
(23)


Similarly we define 
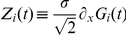
 so that
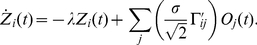
(24)


Finally, we can write our approximation to the ideal observer as
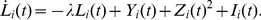
(25)


For this approximation to work, it is crucial that 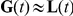. To ensure this condition to hold, we look at the squared distance between 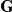 and 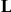 and only let those neurons fire a spike, which add a kernel to 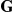 that moves it closer to 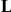. Mathematically this means that a spike is fired if
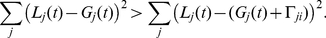
(26)


We can develop the squares in equation (26) to rewrite the spiking criterion as
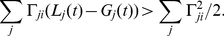
(27)


We define the left hand side of this equation as the membrane potential 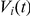 of neuron 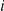. The temporal evolution of 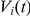 below threshold can be obtained by combining equations (25), (23), (24) and (20) with the left hand side of equation (27). It is then straight forward to find the final result
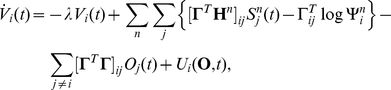
(28)where neuron 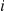 fires a spike if 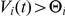, with threshold 
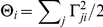
. After firing a spike 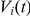 is reset to 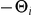.

The dynamics of the slow currents 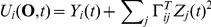 are given by
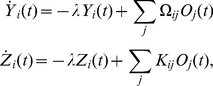
(29)with weights 
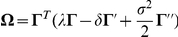
 and 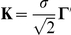.

### Decoding

Decoding in our model reduces to a simple leaky integration of output spikes according to equation (3) of the main text. We can either assume that kernel 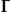 is known a-priori or we can learn it from the output tuning curves, 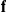, and covariance matrix, 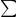 using the relation [6]:
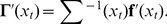
(30)


The two methods give virtually identical results. All results reported in this paper use learnt kernels.

On every trial, we measure the mean and variance of the posterior that we decode from the output spike patterns. The estimator of the stimulus mean, 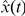 is its expected value: 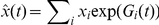. Its variance, 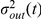 is computed as the second mode of the output posterior, i.e. 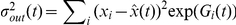.

We measure coding accuracy over many trials as the variance, 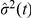, of the stimulus mean 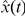 around the real value 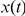. Notice, that variance of the estimator should equal the posterior variances averaged over many trials, i.e. 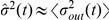, where 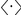 denotes average over trials. For simplicity, we only report the performance measured by 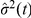.

### Measuring predictability

We will use an indirect measure to assess the predictability of the response of a neuron 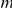 conditioned on the spike trains recorded from a subpopulation of 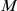 neurons. Let us define the “predicted membrane potential” 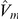 of neuron 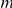 as
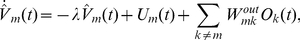
(31)where the sum runs over all recorded neurons and 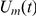 is given by
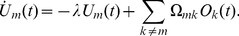
(32)


The predicted membrane potential depicts the total external “driving force” that neuron 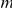 receives from the 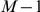 other neurons in the subpopulation. Neurons are generally strongly driven by external input right before they spike. Thus, a high predicted membrane potential and hence a high driving force is an indicator for an enhanced firing probability. We use this intuition to define the predictability, 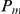, of the activity of neuron 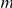 on a given trial as
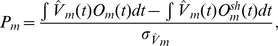
(33)where 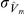 is the standard deviation of 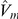 over the entire duration of the trial and 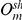 denotes a shuffled version of spike train 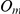. Thus, the predictability 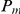 measures the difference between the spike-triggered predicted membrane potentials computed from the recorded spike train and a random spike train with the same number of spikes. Normalizing by 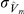 turns 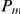 into something like a signal-to-noise ratio.

### Encoding accuracy of the stochastic network

Here we derive an expression for the accuracy with which the stochastic network of section “Comparison to a rate model” can encode the underlying stimulus. The encoding accuracy of this network is limited by two factors: the initial accuracy with which the stimulus is encoded in the input populations and the additional uncertainty that stochastic spike generation adds on top of it.

The input accuracy is determined by the Cramer-Rao bound, 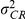, which corresponds to the variance of an optimal estimator. It is related to the Fisher information in the inputs. For the case of uniformly arrayed tuning curves and Poisson firing statistics (as is the case for the input populations), Fisher information, 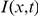, after 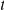 seconds of integration, can be calculated as [55]: 
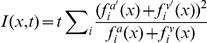
. The Cramer-Rao bound is then given as the inverse of Fisher information, 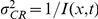.

The output neurons in the stochastic network fire Poisson spikes from a rate, 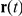, that corresponds to the sum of input spike counts scaled by gain factor K:
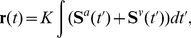
(34)


This corresponds to a mean rate 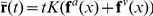. It is obvious, that an optimal estimator of the Poisson spike trains generated from these rates would have a variance of 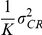.

The noise in input and output spike generation is independent from each other. The variances of input and output estimators therefore add up and we find that the accuracy of an optimal observer of the stochastic output spike trains is given as 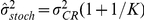.

### Simulation details

The network structure is outlined in figure 1B. Each neural layer contains 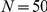 neurons. Input tuning curves are circular Gaussians. For neuron 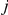 it would take the form 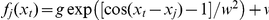 where the preferred direction 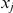 is given by 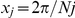. We use 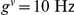, 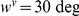 and 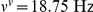 for the visual input and 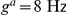, 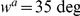 and 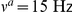 for the auditory input population. The only exception is the simulation of the stochastic network (figure 8B), where we use identical tuning curves in the two inputs 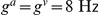, 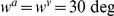 and 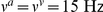.

The input kernels are given by the log tuning curves: 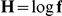. Since we are interested in the log posterior up to an additive constant only, we are free to add or subtract a constant from the kernels. We therefore shift the input kernels, such that 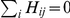. In this way, each input spike adds on average zero to the log posterior 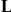. A direct consequence of this shift is that the bias term 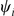 (see eq. 7) equals zero and hence disappears.

The output kernel 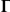 is also chosen to be a circular Gaussian with 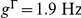, 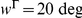 and 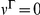. For figures 3E and 4B–4E we used 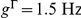 whereas all other parameters remained the same. In accordance with the input kernels, the baseline of 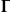 is set such that 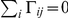.

Parameters for the stimulus dynamics are 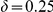 and 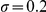. These full dynamics are used in figure 2 and 3. Figure 5 only uses the diffusion and the other figures use static stimuli. The neural leak is set to 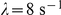.

In order to change the reliability of the input cues (for the simulation in figures 4A and 5C), we multiply the tuning curve of the input neurons in a population by a constant 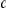. This changes the Fisher information contained in this population by the multiplicative factor 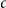: 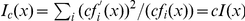. The Cramer-Rao bound of an optimal estimator is therefore divided by 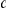. Notice that the input kernels and therefore the feed-forward weights remain unchanged by this operation.

To test the robustness of our network to noise, we add a Gaussian white noise term to the membrane potential: 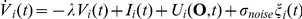, where 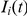 denotes the spiking input to neuron 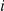 and 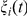 is a white noise term with unit variance, 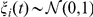. Our simulations are done with noise strengths of 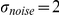, 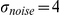 and 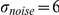.

The differential equations of the membrane potentials are integrated using an Euler method with time step 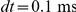. As neighboring output neurons get highly similar input, it is often the case that various neurons cross their spiking threshold in the same time step 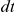. If this happens, we determine which neuron would cross the threshold first assuming a linear voltage increase during the interval 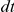. We then let this neuron spike and reset its neighbors. Should there still be a neuron above threshold after this reset, we let it spike as well and so forth until no more neuron is above threshold. We then continue to the next integration step. In most cases however, only one neuron will spike per time interval 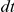.
